# Epidemiological Characteristics and Predisposing Factors for Surgical Site Infections Caused by Bacterial Pathogens Exhibiting Multidrug-Resistant Patterns

**DOI:** 10.3390/antibiotics10060622

**Published:** 2021-05-24

**Authors:** Abdikarim Hussein Mohamed, Hussein Ali Mohamud, Ebubekir Arslan

**Affiliations:** Mogadishu Somalia Turkish Training and Research Hospital, Mogadishu 2526, Somalia; husseinalimohamud@gmail.com (H.A.M.); ebuarslan@hotmail.com (E.A.)

**Keywords:** surgical site infection, antimicrobials, antimicrobial resistance, multidrug-resistant microorganisms

## Abstract

Background: Surgical site infection is the most common kind of nosocomial infection in developed and developing countries. Objectives: Our aim was to identify the prevalence of factors predisposing to multidrug resistance and the antimicrobial susceptibility profile of pathogens. Method: This retrospective study enrolled 10,878 patients who underwent operations in 2018–2020. Pathogens were identified using eosin methylene blue agar. Mueller–Hinton agar was used to assess antimicrobial sensitivity and resistance. In total, 382 patients with confirmed surgical site infection (SSI), whose culture showed growth, were included in the study. Results: The prevalence of SSI in the current study was 3.5%. *Escherichia coli* was the predominant pathogen (35.8%), followed by *Staphylococcus aureus* (21.8%). Antibiotic use, chronic renal failure, diabetes, and emergency operations were found to increase the likelihood of multidrug resistance (OR = 6.23, CI = 1.443–26.881, *p* = 0.014; OR = 5.67, CI = 1.837–19.64, *p* = 0.02; OR = 2.54, CI = 1.46–7.35, *p* = 0.03; OR = 1.885, CI = 1.067–3.332, *p* = 0.002, respectively). The pathogens showed different levels of antimicrobial resistance against ceftriaxone (72.7%), ciprofloxacin (46.6%), and gentamicin (34%). Antimicrobial resistance of about 1–3.4% was exhibited by linezolid, tigecycline, and teicoplanin. Conclusion: The study presented significantly increased multidrug-resistant (MDR) Enterobacteriaceae pathogens isolated from surgical sites. They involve significant morbidity and mortality rates and increased health-related costs.

## 1. Introduction

Surgical site infections (SSIs) are postoperative infections occurring within 30 days of surgery. They involve significant morbidity and mortality rates and increased health-related costs [1,2]. SSI is the most common form of nosocomial infection in both developed and developing countries. The most common causative pathogens are *Escherichia coli (E. coli)*, *Staphylococcus aureus (S. aureus)*, *Klebsiella pneumoniae (K. pneumoniae)*, and *Enterococcus* spp. [3].

The prevalence of surgical site infections varies between 1 and 18% in some developing countries [4]. To date, there have been no studies regarding SSI reported from Somalia.

The increasing number of multidrug-resistant (MDR) microorganisms isolated from surgical sites is an increasing health threat and a public health issue. A multidrug-resistant microorganism is a pathogen that is resistant to 2 or more of the 40 antimicrobial agents. One of the most common reasons for widespread antimicrobial resistance is the poorly controlled use of wide-spectrum antibiotics [4]. Patients with MDR pathogens are resistant to most first-line antimicrobial classes. This resistance necessitates the handling of these patients with a prolonged hospital stay. It involves increased healthcare costs and admissions to intensive care units and significant morbidity and mortality.

The patient-related risk factors for SSI include advanced age, obesity, current infection, diabetes mellitus, low albumin, and smoking. Surgery-related risk factors include prolonged and emergency operations, inadequate surgical scrubbing and skin preparation, abdominal surgeries, and contamination of the contents. Trauma, shock at the time of admission, the requirement of blood transfusions, hypothermia, hypoxia, and elevated blood glucose levels are physiological conditions that enhance the risk of SSIs [5].

In addition to addressing the increasing rate of SSIs due to multidrug-resistant pathogens, the importance of this study is its identification of the factors determining the widespread distribution of MDR pathogens and its evaluation of prevalence, the most common etiological pathogens, and the antimicrobial susceptibility pattern.

## 2. Methods

This study enrolled 10,878 patients who underwent operations at Mogadishu Somali Turkey Reccep Tayyip Erdogan Training and Research Hospital whose medical records were retrospectively reviewed between 2018 and 2020. The applied definition of SSI was described by the Centers for Disease Control and Prevention (CDC). In total, 382 patients with confirmed surgical site infection whose cultures showed growth were included in the study.

### 2.1. Inclusion and Exclusion Criteria

All patients who underwent surgeries that developed SSI less than 30 days after the operation were included in the study. All wounds not related to operations or abscesses were excluded from the study.

### 2.2. Sample and Culture

Surgical site drainage cultures and antibiograms were performed by the laboratory and microbiology unit of the hospital via standard laboratory protocols, and the findings of these tests were used for the study. After the surgical site drainage cultures were obtained, identifying the bacterial pathogens was achieved by a combination of BD BBL crystal identification system, as well as biochemical tests, including oxidase and citrate test, coagulase test, and catalase test according to the system of the Clinical and Laboratory Standards Institute (CLSI). Subsequently, antimicrobial sensitivity tests were performed using the standard Kirby–Bauer disk diffusion method and commercial disks (Oxoid discs). The ESBL were identified from positive cultures using cephalosporins (cefuroxime + ceftriaxone) and amoxicillin-clavulanic acid as the identifying disc to differentiate the patterns of growth between antibiotics with lactamase inhibitor and those without. *Enterococcus* antimicrobial sensitivity and resistance were assessed using blood agar. Cefoxitin-resistant *Staphylococcus aureus* was assessed in the same way as methicillin-resistant *Staphylococcus aureus* (MRSA).

Antimicrobial susceptibility testing (AST) were identified by the disk diffusion zone of growth inhibition and MIC values (susceptible (S) intermediate (I), and resistant (R) categories) according to the CLSI criteria except for breakpoints for tigecycline where used FDA minimum inhibitory concentrations (MICs) breakpoints for susceptible (≤2 mg/L), intermediate (4 mg/L), and resistant (≥8 mg/L). The antibiotic susceptibility of the pathogens was studied by using piperacillin/tazobactam 100/10 mcg, meropenem 10 mcg, ertapenem 10 mcg, colistin 10 mcg, amikacin 30 mcg, tigecycline 15 ug, cefepime 30 ug, cefazolin 30 ug, ceftazidime 30 ug, piperacillin 100 ug, linezolid 30 mcg, clindamycin 2 mcg, penicillin G 1 U, trimethoprim/sulfamethoxazole 1.25/23.75 mcg, vancomycin 30 mcg, daptomycin 30 mcg, tetracycline 30 mcg, erythromycin 15 mcg, cefoxitin 30 mcg, ciprofloxacin 5 mcg, nitrofurantoin 300 mcg, and teicoplanin 30 ug.

### 2.3. Sample Size

In total, 382 patients with confirmed SSIs whose cultures showed growth were included in the study. The patients were grouped into five age categories: newborn to 18 years, 19–39 y, 40–58 y, 59–76 y and >77 y.

### 2.4. Predisposing Factors for Multidrug Resistance

The study assessed factors affecting the incidence of multidrug resistance of microorganisms, divided into two groups: The patient-related factors included the age and gender of the patient, antimicrobial use, and coexisting conditions (diabetes, chronic renal failure, and obesity). The surgery-related factors included the type of surgeries (emergency and elective operations), the site of admission (non-ICU patients and ICU patients), type of patient (trauma or non-trauma), and intraoperative and postoperative blood transfusions.

### 2.5. Ethical Approval and Informed Consent

All patients gave their informed consent. The ethics approval form was received from the ethics committee of Mogadishu Somali Turkey Reccep Tayyip Erdogan Training and Research Hospital (REF. MSTH-2900).

### 2.6. Statistical Analysis

Descriptive and inferential measures were employed for the data analysis. As regards the categorical variables, the frequencies and proportions were presented as point estimates. As regards quantitative variables, the mean (±SD) was employed wherever necessary. The confidence interval (95%) was used to derive an interval estimate of the mean for the population. Binary analyses were performed to assess the association between independent and dependent variables. The odds ratios (ORs) and their 95% confidence intervals (CIs) were calculated. A *p* value of <0.05 was considered statistically significant. Variables that showed statistical significance under the bivariate analysis were included in the multivariate logistic regression model to determine factors that contribute to multidrug resistance. The adjusted ORs with their 95% CIs were computed to determine the association. All statistical analyses were performed using the Statistical Package for Social Sciences (SPSS -IBM) for Windows version 23.

## 3. Results

Of 10,878 patients, 382 patients displayed confirmed surgical site infections and cultures showing growth. These patients comprised the study population. The prevalence of surgical site infection in the current study was 3.5%. Almost all patients had experienced an open approach to their various types of surgery. All patients received cephalosporin antimicrobial prophylaxis before the operation. The mean age of the patients was 34.5 ± 20.6 years.

Antibiotic use, chronic renal failure (CRF) and diabetes were found to increase the chance of multidrug resistance (OR = 6.23, CI = 1.443–26.881, *p* value = 0.014; OR = 5.67, CI = 1.837–19.64; *p* value = 0.02; OR = 2.54, CI = 1.46–7.35, *p* value = 0.03, respectively). Patients who underwent emergency operations were more vulnerable to multidrug-resistant microorganisms compared to patients who underwent elective surgeries (OR = 1.885, CI = 1.067–3.332, *p* = 0.002). However, gender, age, obesity, section of admission, type of patient and experience of transfusion (AOR = 0.732, CI = 0.418–1.282, *p* value = 0.275; AOR = 1.245, CI = 0.961–1.612, *p* value = 0.096; AOR = 2.007, CI = 0.925–4.357, *p* value = 0.078; AOR = 0.617, CI = 0.302–0.938, *p* value = 0.185; AOR = 1.41, CI = 0.84–2.35, *p* value = 0.159; AOR = 1.066, CI = 0.607–1.874, *p* value = 0.824, respectively) were not associated with multidrug resistance (Table 1).

The gender distribution of surgical site infections was 65.4% male (250 patients) and 34.6% female (182 patients), which shows a twofold increased predominance of SSIs in males. In total, 81.4% of the patients had a single isolate, and the remaining 18.6 cases showed polymicrobial pathogens. In our study, as illustrated in Table 2, about 34 pathogens were found in the results from cultures that showed growth. *E. coli* was found to be the most predominant pathogen, encountered in 137 patients (35.8%), followed by *Staphylococcus aureus* in 83 patients (21.8%) and *Klebsiella pneumonia* in 49 patients (12.9%). At the same time, 31 of the cases displayed *Acinetobacter baumannii* (8.1% of patients), 27 patients had *Pseudomonas aeruginosa* (7% of the cases), 14 patients had *Proteus mirabilis* (3.7%), *Enterobacter cloacae* was found in 11 patients (2.9%), and 6 of the cases (1.6% of patients) had *Streptococcus* spp. (*Streptococcus pyogenes 2*, *Streptococcus Pneumoniae 2*, *Streptococcus sanguinis 1*, *Streptococcus intermedius 1*).

Other rare pathogens isolated from the cultures included the *Staphylococcus* species (*Staphylococcus epidermidis*, *Staphylococcus* saprophyticus, *Staphylococcus hominis*, *Staphylococcus haemolyticus*, and *Staphylococcus intermedius*), *Pantoea agglomerans*, *Enterococcus faecium*, *Corynebacterium* spp., *Acinetobacter lwoffii*, *Providencia stuartii*, *Providencia rettgeri*, *Citrobacter freundii*, *Morganella morganii*, *Enterobacter aerogenes*, and the *Proteus vulgaris* group.

We also assessed the presence of extended-spectrum beta-lactamases (ESBL)-producing pathogens. ESBL-producing pathogens were found in 15% of the cases (57 patients). *E. coli* was the most common pathogen, arising in 31 patients (54.5%). Concerning the overall *E. coli* isolates, 22.6% of the *E. coli* found produced the extended-spectrum beta-lactamases (ESBL), which was a significant increase. *Klebsiella pneumonia* was identified to be the second most common pathogen producing ESBL. It appeared in 19 patients (33.3%), followed by *Proteus mirabilis* in 3 patients (5.2%).

The prevalence and the pattern of methicillin-resistant *Staphylococcus aureus* (MRSA)-producing species were evaluated throughout the study. The prevalence of MRSA was 3.1% (12 patients). In total, 9/83 *S. aureus* isolates produced MRSA (10.8%). The three remaining kinds of MRSA were produced by *Staphylococcus* *epidermidis*, *Staphylococcus saprophyticus*, *and Staphylococcus hominis*.

The antibiotic sensitivity and resistance of the pathogens were assessed, and the antimicrobial profiles are comprehensively depicted in Table 3.

The Gram-negative pathogens displayed antimicrobial resistance levels of about 65.5% against cephalosporins, 52% against fluoroquinolones, 30% against aminoglycosides, and 14.6% against carbapenems. The Gram-positive pathogens revealed antimicrobial resistance levels of about 36.3% against cephalosporins, 22.8% against fluoroquinolones, 12.7% against aminoglycosides, and 7% against carbapenems.

As regards individual antibiotic-resistant patterns, ampicillin was the most resistant antibiotic (92.2%), followed by cefotaxime (81%), cefuroxime (76.6%), and cotrimoxazole (74.9%). The antibiograms revealed that 72.7% of the identified pathogens were resistant toward ceftriaxone, 46.6% to ciprofloxacin, 34% toward gentamicin, 48.1% to amoxicillin-clavulanic acid, 13.9% to amikacin, and 10.2% toward clindamycin. About 18–27% of the pathogens were resistant to cefoperazone–sulbactam, piperacillin–tazobactam, and ampicillin–sulbactam.

In our study, antimicrobial resistance levels of about 1–3.4% were revealed by linezolid, tigecycline, and teicoplanin, respectively. Vancomycin and daptomycin did not show any antimicrobial resistance, including methicillin-resistant *Staphylococcus aureus* (MRSA) and *Enterococcus*. Carbapenems revealed a high sensitivity rate against pathogens of about 85.9% (86.8% to imipenem, 86.6% to ertapenem, and 82.9% to meropenem).

*E. coli* was the most common cause of surgical site infection in our retrospective study. *E. coli* was predominantly resistant to ampicillin (93.2%), cephalosporins (61.9%), and fluoroquinolones (53.5%). *Klebsiella* showed greater resistance toward cephalosporins (70%), ampicillin (97.7%) and fluoroquinolones (about 62.3%), compared to *E. coli. S. aureus* had the highest resistance rate against fluoroquinolones in 70.4% of the cases.

The spectrum of resistance to carbapenems was assessed; *pseudomonas* revealed the highest resistance to carbapenems at 13%, followed by *Acinetobacter* at 11.3% and *Klebsiella* at 8.8%.

Of 382 patients, 81.7% showed a multidrug resistance pattern, while 14.9% of the patients showed extensive drug resistance (XDR). *Acinetobacter baumannii* revealed the highest MDR and XDR spectra (100%), followed by *E. coli* at 96.4% and *Klebsiella pneumonia* at 73.5%.

## 4. Discussion

Multidrug-resistant (MDR) pathogens isolated from surgical sites have increased in number. They are a major health threat and public health issue. A systematic review and meta-analysis performed by Martin et al. suggests that diabetes is a significant risk factor for SSIs, potentially due to the vascular changes and leukocyte dysfunction it induces beyond its role in causing hyperglycemia [6]. A large cohort study of 4690 patients who underwent general surgical procedures concluded that emergency surgery, prolonged operating times, surgical site, prolonged preoperative hospital stay, higher ASA score, dirty/infected wound class, the presence of surgical drains, and intraoperative transfusion were independent risk factors for SSI (Isik O et al. p 0.05) [7]. The four independent parameters that increase the risk of SSI, reported by the Study on Efficacy of Nosocomial Infection Control (SENIC) trial, were abdominal operations, prolonged operations (>2 h), a contaminated or dirty procedure, and the presence of more than three diagnoses at the time of discharge [5].

Besides the increasing rate of SSIs due to MDR pathogens and the scarcity of literature regarding factors predisposing patients with SSIs to MDR, this study intended to identify factors determining the widespread distribution of MDR microorganisms. Our study revealed that antibiotic use, emergency operations, diabetes, and chronic renal failure are significantly related to multidrug resistance. The antimicrobial treatment of chronic renal failure patients with surgical site infections is challenging. The use of antibiotics should be adjusted according to renal function, the appropriate empirical therapy, and the culture test results once available to minimize the worsening of preexisting antimicrobial resistance. Age, obesity, trauma, and intraoperative blood transfusion are significantly associated with SSIs. However, the current study revealed they were not associated with multidrug resistance patterns against microorganisms [5,7]. Preoperative antibiotic prophylaxis, antiseptic skin preparation, hair removal, the avoidance of hypothermia, and perioperative glycemic control compose the bundle approach aimed at reducing SSIs to improve safety.

The constant assessment of the changing trends, besides pathogens and their antibiotic sensitivity and resistance patterns, is particularly crucial in developing countries where SSI prevalence and morbidity and mortality rates are high [8]. To date, there have been no studies into SSIs performed in Somalia. The prevalence of surgical site infection in our study was 3.5%, and a similar prevalence rate was found in other studies [5,9].

The pattern of antimicrobial sensitivity and resistance differed in the different classes of antibiotics and in the pathogens isolated from the culture. *E. coli* was the predominant pathogen for all age groups in our study and topped the table (35.8%), which coheres with other studies [6,10,11]. Research performed by S Elgoharia and associates reported that significantly increased *Enterobacteriaceae* pathogens cause SSIs. Another notable finding in our study is that *Staphylococcus aureus* was related to a significant reduction (21.8%) compared to *E. coli*, which agrees with the results of S Elgoharia and colleagues reporting similarly [10]. A review article by Lindsey M. and associates discussed the pattern of antimicrobial resistance toward pathogens and revealed *E. coli* to be the predominant pathogen in their study [12]. There is variation in the spectrum of pathogens causing SSIs. A prospective cohort study from Rwanda by Marie Josée and partners reported on the common pathogens in surgical site infection and identified that *Klebsiella* was the predominant pathogen, followed by *E. coli* [4].

Males were more vulnerable to surgical site infection in our study by nearly double (65.4%). The mean age of patients in our study was 34.5 years, which contrasts with other studies [13,14].

Second- and third-generation cephalosporins are the most common antibiotic classes prescribed for antimicrobial prophylaxis, prescribed in most admitted patients. However, they have the highest resistance rate against pathogens. Correlations between early culture and antibiotic results are necessary for deteriorating patients once culture results are available [4].

*E. coli* was predominantly resistant to ampicillin (93.2%) and showed an increasing resistance trend toward cephalosporins (61.9%) and fluoroquinolones (53.5%), which coheres with studies [12]. *Klebsiella* showed a higher resistance pattern toward cephalosporins (70%) and fluoroquinolones (62.3%) than *E. coli*. *S. aureus* had the greatest resistance to fluoroquinolones (70.4%) [15].

The current study reviewed the spectrum of ESBL cases (15%). In total, 23% of *E. coli* isolates (31/137) produced extended-spectrum beta-lactamases, which mirrors national surveillance data (23%) reported from Japan by Yoshio Takesue and associates [3]. *E. coli* was the highest in terms of ESBL, followed by *Klebsiella*.

Carbapenem-resistant Enterobacteriaceae (CRE) isolates have increased in number in recent years. This is a healthcare challenge due to the associated morbidity and mortality requiring complex management because of the extensive drug resistance pattern [16,17]. Carbapenems revealed a high sensitivity rate against pathogens (about 85.9%) in the current study.

John A. and collaborators reported on the causative pathogens and outcomes related to surgical site infection; the authors identified a significant increase (20.6%) in methicillin-resistant *S. aureus* (MRSA), which is related to increased mortality and health-related costs [2]. National surveillance data reported from Japan by Yoshio Takesue and colleagues showed a significant reduction in methicillin-resistant *S. aureus*, from 72.5% to 53.8% [3]. Methicillin-resistant *S. aureus* (MRSA) was less prevalent in our study compared to the above studies. MRSA-producing *Staphylococcus aureus* was found in 10.8% of the cases, but all cases were sensitive to vancomycin. Vancomycin and daptomycin did not show any antimicrobial resistance, including methicillin-resistant *Staphylococcus aureus* (MRSA) and *Enterococcus*.

The limitations of our study include that it used a retrospective design that missed some variables influencing the multidrug resistance pattern, including intraoperative abdominal contaminations. Second, the study was single-center-based. This is the first and only study from Somalia. Further prospective studies are needed to afford detailed insight into the variables affecting the multidrug resistance and extensive drug resistance spectra.

## 5. Conclusions

The study results showed significantly increased multidrug-resistant (MDR) Enterobacteriaceae pathogens isolated from surgical sites, and they involve significant morbidity and mortality rates and increased health-related costs. *Acinetobacter baumannii* revealed the highest multidrug resistance and extensive drug resistance spectrum; tigecycline has shown a higher sensitivity rate against the pathogen. Antibiotic use, emergency operations, diabetes, and chronic renal failure are significantly related to multidrug resistance patterns. Antimicrobial prophylaxis, appropriate skin preparation and hair removal, avoidance of hypothermia, and perioperative glycemic control are part of the bundle approach to reduce SSIs to improve safety.

## Figures and Tables

**Table 1:** Comprehensive analysis of risk factors predisposing to multidrug-resistant microorganisms causing surgical site infection.

**Patient-Related Factors**	**MDR**		**AOR**	**95% CI**	***p* Value**
	**Yes**	**No**			
Age			1.245	0.961–1.612	0.096
Newborn–18 y	66	21			
19–39 y	132	27			
40–58 y	60	15			
59–76 y	40	6			
>77 y	13	1			
Gender			0.732	0.418–1.282	0.275
Male	202	48			
Female	110	22			
Diabetes	61	4	2.54	1.46–7.35	0.03
Chronic renal failure	38	3	5.67	1.837–19.64	0.02
Obesity	29	11	2.007	0.925–4.357	0.078
Antibiotic use	156	24	6.23	1.443–26.881	0.014
**Surgery-Related Factors**	**MDR**		**AOR**	**95% CI**	***p* Value**
	**Yes**	**No**			
Type of Surgery			1.885	1.067–3.332	0.002
Elective	89	34			
Emergency	223	36			
Section of Admission			0.617	0.302–0.938	0.185
Non-ICU	218	55			
ICU	93	16			
Type of patient			1.41	0.84–2.35	0.159
Non-trauma patients	149	46			
Trauma patients	125	62			
Intra- and postoperative blood transfusions	57	17	1.066	0.607–1.874	0.824

AOR—adjusted odds ratio, CI—confidence interval = 95%; a *p* value < 0.05 is significant.

**Table 2:** Most common pathogens.

Type of Microorganisms	No. Patients	Percentage
*E. coli*	137	35.8%
ESBL-producing *E. coli*	31	
MDR		
Yes	132	96.4%
No	5	3.6%
*Staphylococcus aureus*	83	21.8%
*MRSA*	9	
MDR		
Yes	57	68.7%
No	26	31.3%
*Klebsiella pneumonia*	49	12.9%
ESBL-producing *Klebsiella*	19	
MDR		
Yes	36	73.5%
No	13	26.6%
*Acinetobacter baumannii*	31	8.1%
MDR		
Yes	31	100%
No	0	0%
*Pseudomonas aeruginosa*	27	7%
MDR		
Yes	13	48%
No	14	52%
*Proteus mirabilis*	14	3.7%
ESBL-producing *Proteus*	3	
*Enterobacter cloacae*	11	2.9%
ESBL-producing *Enterobacter*	2	
*Streptococcus* spp.	6	1.6%
*Pantoea agglomerans*	3	0.8%
*Pseudomonas oryzihabitans*	2	0.5%
*Stenotrophomonas maltophilia*	2	0.5%
Others	17	4.4%
ESBL	2	
MRSA	3	
Total	382	100.0%

**Table 3:** Distribution of antibiotic resistance and antimicrobial profiles of specific pathogens.

Medications	Total/Resistant	*E. coli*	*Klebsiella*	*Pseudomonas*	*Acinetobacter*	*S. aureus*
Cefazolin	137 (72.3%)	69.5%	73.7%	58.8%	88.7%	77.9%
Cefotaxime	58 (81.0%)	80.6%	93.3%	90%	90.9%	88%
Cefoxitin	223 (43.9%)	48%	67.9%	75%	70.6%	55.3%
Cefuroxime	201 (76.6%)	71.9%	80.0%	80%	79.2%	84.1%
Ceftriaxone	128 (72.7%)	67.2%	78.9%	70%	76.9%	77.4%
Ceftazidime	101 (60.4%)	46.7%	60.0%	75%	75%	57.1%
Cefixime	137 (73.7%)	74.7%	89.3%	92.3%	93.8%	87.8%
Ampicillin	219 (92.2%)	93.2%	97.7%	100%	100%	97.3%
Cefoperazone–sulbactam	118 (27.1%)	14.3%	15.0%	30%	27.3%	13.2%
Piperacillin–tazobactam	118 (27.1%)	19%	16.7%	20%	20%	25%
Amoxicillin clavulanic acid	239 (48.1%)	45%	57.4%	61.5%	60%	56.8%
Gentamicin	338 (34.0%)	38.8%	28.9%	33.3%	33.3%	39.1%
Amikacin	245 (13.9%)	8.7%	4.9%	4.5%	3.8%	9.9%
SMX–TMP	339 (74.9%)	78%	75%	76.9%	73.3%	81.5%
Ciprofloxacin	290 (46.6%)	58.5%	69.4%	52.9%	60%	76.2%
Levofloxacin	137 (36.5%)	37.1%	47.1%	41.7%	46.2%	40%
Imipenem	250 (13.2%)	1.7%	2.5%	5%	4.2%	1.4%
Meropenem	123 (17.1%)	9.1%	22.2%	25%	25%	18.2%
Ertapenem	142 (13.4%)	8.8%	13.3%	16.7%	14.3%	10.4%
Linezolid	96 (1.0%)	0%	0%	0%	0%	1.5%
Vancomycin	95 (0%)	0%	0%	0%	0%	0%
Tigecycline	74 (2.7%)	0%	0%		0%	7.4%
Colimycin	45 (6.7%)	0%		11.1%	0%	33.3%
Teicoplanin	29 (3.4%)		0%			5%
Daptomycin	54 (0%)					
Clindamycin	88 (10.2%)	0%		0%	50%	7%
Tobramycin	13 (69.2%)	50%		50%	100%	
Ampicillin–sulbactam	28 (17.9%)	100%				12%
Quinupritin–dalfopristin	38 (0%)	0%			0%	0%
Oxycycline	51 (21.6%)	0%		0%		22.5%
Tetracycline	87 (42.5%)	66.7%		50%		43.8%

## Data Availability

Data included in the manuscript.

## Abbreviations

CDC: Centers for Disease Control and Prevention; CRF: chronic renal failure; EMB: eosin methylene blue agar; ESBL: extended-spectrum beta-lactamases; MDR: multidrug-resistant; MRSA: methicillin-resistant *Staphylococcus aureus*; SSI: surgical site infection; XDR: extensive drug-resistance.
